# Fatal early-onset checkpoint inhibitor pneumonitis in a patient with advanced squamous-cell lung cancer with underlying pulmonary fibrosis: a case report and review of the literature

**DOI:** 10.3389/fonc.2025.1672093

**Published:** 2025-11-04

**Authors:** Daria M. Keller, Dagobert Żarczyński, Anna Rybacka, Barbara Kuźnar-Kamińska

**Affiliations:** ^1^ Department of Pulmonology, Allergology and Pulmonological Oncology, Poznan University of Medical Sciences, Poznań, Poland; ^2^ 1st Department of Cardiology, Poznan University of Medical Sciences, Poznań, Poland; ^3^ Department of Diagnostic Imaging, Poznan University of Medical Sciences, Poznań, Poland

**Keywords:** checkpoint inhibitor pneumonitis, idiopathic pulmonary fibrosis, pembrolizumab, lung cancer, immune-related adverse event

## Abstract

**Introduction:**

We report a case of fulminant checkpoint inhibitor pneumonitis (CIP) occurring after a single dose of immune checkpoint inhibitor (ICI) therapy in a patient with advanced non-small cell lung cancer and underlying fibrotic interstitial lung disease (ILD), illustrating a rare but clinically significant and often fatal immune-related adverse event.

**Main symptoms and clinical findings:**

A 78-year-old woman with stage IVb squamous-cell carcinoma of the lung and idiopathic pulmonary fibrosis (IPF) developed progressive dyspnea, hypoxemia, and systemic inflammation shortly after receiving her first dose of pembrolizumab. High-resolution computed tomography (HRCT) revealed new bilateral ground-glass opacities superimposed on a fibrotic background with a definite usual interstitial pneumonia (UIP) pattern.

**Diagnoses, interventions, and outcomes:**

Fulminant early-onset CIP was diagnosed after exclusion of infectious causes. Despite discontinuation of immunotherapy and escalation of immunosuppressive treatment—including high-dose corticosteroids, mycophenolate mofetil, and infliximab—the patient’s respiratory status deteriorated, resulting in death 27 days after treatment initiation.

**Conclusion:**

This case illustrates that life-threatening CIP can occur after a single dose of ICI in patients with fibrotic ILD. It emphasizes the urgent need for risk-adapted treatment strategies and enhanced monitoring protocols in this high-risk population.

## Introduction

Immune checkpoint inhibitors (ICIs) have revolutionized the treatment of a broad spectrum of malignancies, offering durable antitumor responses and improved survival outcomes. In non-small cell lung cancer (NSCLC), especially among patients with high programmed death-ligand 1 (PD-L1) expression, ICIs such as pembrolizumab have become standard-of-care in both frontline and relapsed settings. However, these benefits are tempered by a range of immune-related adverse events, of which checkpoint inhibitor pneumonitis (CIP) is among the most serious and potentially fatal (1).

The reported incidence of CIP varies widely, ranging from 2.7% to 20%, with a substantial proportion of cases classified as severe (grade ≥3) according to the Common Terminology Criteria for Adverse Events (CTCAE), version 5.0. Mortality estimates for high-grade CIP range from 12.8% to 22.7%, underscoring the clinical significance of this complication (2, 3). Although CIP typically emerges within 2 to 3 months following initiation of anti–PD-1 or PD-L1 therapy, the time to onset can vary substantially, from as early as several days to as late as two years (4, 5). Notably, early-onset CIP, defined as occurring within the first 6 weeks of therapy, is increasingly recognized and appears to be associated with more severe presentations and poorer outcomes (6).

The clinical manifestations of CIP are heterogeneous, ranging from subtle symptoms such as dry cough and mild exertional dyspnea to acute hypoxemic respiratory failure. Some patients may remain asymptomatic and are diagnosed incidentally based on new pulmonary opacities observed on surveillance imaging. Diagnosing CIP is particularly challenging due to its nonspecific symptoms and significant overlap with other pulmonary conditions, including infectious pneumonia, tumor progression, radiation pneumonitis, aspiration, or drug-induced lung injury (7–9). This diagnostic ambiguity often delays treatment and contributes to morbidity and mortality.

Identifying predisposing risk factors is essential for early recognition and personalized management. In patients with interstitial lung disease (ILD), especially idiopathic pulmonary fibrosis (IPF), chronic epithelial injury and baseline immune dysregulation contribute to a fibrotic, immunologically primed lung environment. This state is characterized by ongoing fibroblast activation, extracellular matrix deposition, and aberrant cytokine signaling, including elevated IL-6, IL-17a, and IL-35 and impaired regulatory T-cell activity (2, 10). These pathological features may increase the risk of developing CIP and contribute to more rapid disease progression following ICI treatment. In a retrospective cohort of NSCLC patients treated with ICIs, Cho et al. found that the presence of preexisting ILD was significantly associated with an increased risk of developing CIP (11). Atchley et al. similarly found that radiographic evidence of fibrosis was associated with markedly elevated CIP risk (12). In an extensive case-control study, Deng et al. identified ILD, emphysema, and pleural effusion as independent predictors of severe CIP. They developed a validated risk-scoring tool incorporating these variables (13).

In this report, we present the case of a 78-year-old woman with advanced squamous-cell carcinoma of the lung and underlying IPF who developed fulminant CIP just 12 days after her first dose of pembrolizumab. Her case underscores the potentially devastating course of early-onset CIP in high-risk individuals. It illustrates the need for careful pre-treatment stratification, close post-treatment monitoring, and consideration of alternative therapeutic strategies in patients with fibrotic ILD.

## Patient information

A 78-year-old woman was diagnosed in March 2025 with stage IVb squamous-cell carcinoma of the left lung. She was a lifelong nonsmoker. Her past medical history included well-controlled hypertension, type 2 diabetes mellitus, and paroxysmal atrial fibrillation. As part of her oncologic staging, a contrast-enhanced computed tomography (CT) performed on April 18, 2025, revealed a large tumor in the left lower lobe with mediastinal and supraclavicular lymphadenopathy. Additionally, fibrotic changes in the lung parenchyma — including subpleural and basally predominant reticulations, lower lobe volume loss, and honeycombing — were consistent with a definite UIP pattern. This led to a concurrent diagnosis of IPF. The diagnosis of IPF was confirmed by a multidisciplinary team, including a pulmonologist and a thoracic radiologist. An experienced oncologist also participated in the decision as part of the systemic treatment qualification process. A left adrenal nodule was noted as suspicious for metastasis. Immunohistochemistry confirmed PD-L1 expression >50%. Pulmonary function tests were not performed, as the patient was referred from another center and systemic therapy was prioritized; the radiologic UIP pattern was considered sufficient to guide management. Pembrolizumab monotherapy (200 mg every three weeks) was initiated as first-line treatment. At baseline, the patient’s oxygen saturation (SpO_2_) on room air was 94%.

## Clinical findings

Two weeks after initiating immunotherapy, the patient developed dyspnea, low-grade fever, and non-bloody diarrhea. The next day, she experienced a syncopal episode, prompting emergency evaluation and initiation of home oxygen therapy. Despite supportive care, her symptoms worsened, and she was admitted to the hospital with severe hypoxemia (SpO_2_ 60% on room air). On physical examination, she was tachypneic with fine bibasilar crackles. Laboratory results showed elevated white blood cell count, C-reactive protein (CRP), and lactate dehydrogenase, along with anemia, hypoalbuminemia, and a marked rise in neutrophil-to-lymphocyte ratio and platelet-to-lymphocyte ratio (14). These abnormalities are consistent with fulminant CIP and are detailed in **Table 1**. Blood and urine cultures, a multiplex respiratory PCR (polymerase chain reaction) panel, and urinary antigen tests for *L. pneumophila* and *S. pneumoniae* were all negative (**Supplementary Table 1**).

**Table 1:** Laboratory parameters before and after pembrolizumab administration.

Parameter	Normal range (SI units)	Patient’s values – before pembrolizumab infusion (24.04.2025)	Patient’s values – hospitalization due to CIP (15–21.05.2025)
Absolute eosinophil count	0.04–0.40 × 10^9^/L	0.24	0.02 → 0.00
Absolute lymphocyte count	1.0–3.0 × 10^9^/L	2.39	0.82 → 0.8
Absolute neutrophil count	1.5–7.5 × 10^9^/L	5.66	29.97 → 20.73
Albumin	35–50 g/L	Not assessed	30.12 g/L ↓
Alveolar nitric oxide	<6.35 ppb	Not assessed	Not assessed
Blood and urine cultures	Negative	Not assessed	Negative
C-reactive protein	<5 mg/L	2	72.8 → 81.1 mg/L ↑↑
Hemoglobin	7.4–9.9 mmol/L (♀)	7.6	6.4 → 6.3 mmol/L ↓
Lactate dehydrogenase	135–225 U/L	Not assessed	833 → 3585 U/L ↑↑↑
Neutrophil-to-lymphocyte ratio	< 3, ≥6 elevated	2	12 → 26 ↑↑
Platelets	150–400 × 10^9^/L	193	316 → 99
Platelet-to-lymphocyte ratio	<180	80.75	385 → 123.75
Procalcitonin	<0.05 ng/mL (normal); <0.1 low risk for sepsis	0.08	0.1 → 0.13 ng/mL ↑
White blood cells	4.0–10.0 x10^9^/L	8.98	11.47 → 22.64 x10^9^/L ↑↑↑

## Timeline

The sequence of key clinical events is presented in **Table 2**.

**Table 2:** Chronological summary of clinical events.

Date	Event
March 2025	Diagnosis of squamous-cell lung carcinoma
April 18, 2025	Baseline CT revealed lung mass and fibrotic lung changes consistent with IPF
April 24, 2025	First and only dose of pembrolizumab administered (200 mg IV)
May 10, 2025	Onset of new symptoms: dyspnea, low-grade fever, and diarrhea
May 11, 2025	Syncopal episode; emergency evaluation; initiation of home oxygen therapy
May 15, 2025	Hospital admission for worsened hypoxemia (SpO_2_ 60% on room air); oxygen via nasal cannula escalated to face mask
May 16, 2025	Initiation of high-dose intravenous corticosteroids; follow-up HRCT showed new bilateral ground-glass opacities and pleural/pericardial effusions; switched to HFNOT
May 17, 2025	Escalation to noninvasive ventilation
May 19, 2025	Mycophenolate mofetil added to treatment regimen
May 20, 2025	Infliximab added to treatment regimen
May 21, 2025	Patient passed away — 27 days after initiation of pembrolizumab

CT, computed tomography; HFNOT, high-flow nasal oxygen therapy; HRCT, high-resolution computed tomography; IPF, idiopathic pulmonary fibrosis; IV; intravenous; SpO_2_, oxygen saturation.

## Diagnostic assessment

**Figure 1:**
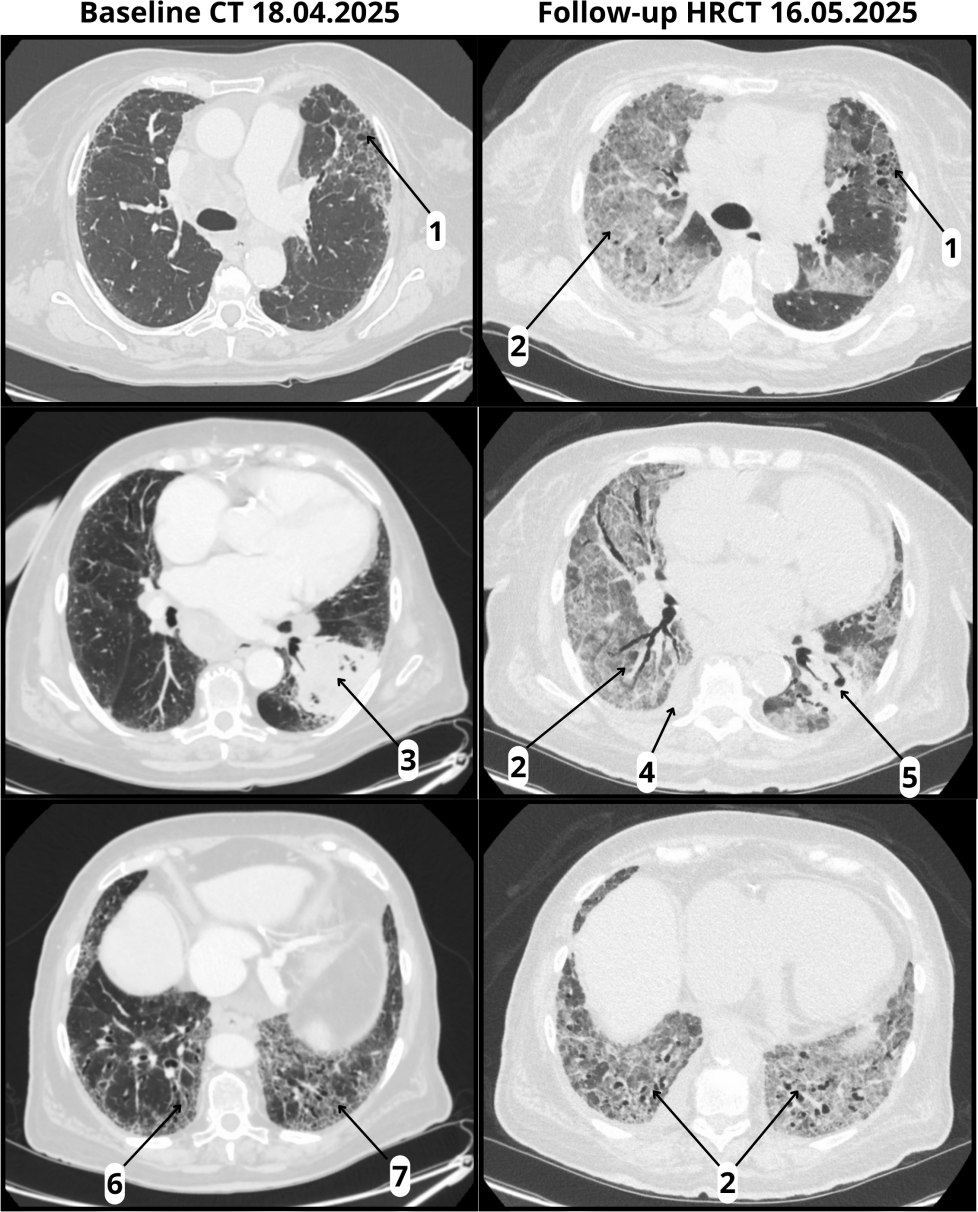
Baseline CT and follow-up HRCT. Baseline CT (18 April 2025) and follow-up HRCT (16 May 2025, performed 22 days after pembrolizumab infusion): (1) Honeycombing; (2) ground-glass opacities (absent at baseline, new at follow-up); (3) tumor in the left lower lobe with pleural involvement; (4) right pleural effusion (new at follow-up); (5) reduction in tumor size (follow-up); (6) traction bronchiectasis; (7) volume loss.

Evaluation included physical examination, laboratory testing, and microbiological studies. Infectious causes were excluded through negative cultures, urinary antigen tests, and multiplex respiratory panel testing. Sputum collection was not feasible because the patient was unable to expectorate, and bronchoscopy with bronchoalveolar lavage was planned but ultimately not performed due to the rapidly deteriorating clinical status. Follow-up high-resolution computed tomography (HRCT) on May 16, 2025, revealed diffuse bilateral ground-glass opacities, a right pleural effusion, and pericardial fluid, all superimposed on a fibrotic background with honeycombing, consistent with a definite UIP pattern. (**Figure 1**). The imaging findings were radiologically compatible with both acute interstitial pneumonia (AIP) and acute respiratory distress syndrome (ARDS). Since AIP refers to idiopathic acute lung injury in patients without pre-existing ILD, and ARDS is a clinical diagnosis requiring both radiologic and clinical criteria, we interpreted the presentation as consistent with ARDS, according to A New Global Definition of Acute Respiratory Distress Syndrome (15). The differential diagnosis included CIP, infectious pneumonia, tumor progression, and aspiration. Given the temporal association with pembrolizumab initiation, rapid clinical deterioration, radiologic features, and negative infectious workup, a diagnosis of fulminant early-onset CIP was established. The patient’s prognosis was considered poor due to the coexistence of IPF and advanced-stage lung cancer.

## Therapeutic intervention

Immunotherapy was discontinued at the time of hospital admission. The patient initially received supplemental oxygen via a nasal cannula, which was escalated to a face mask. As respiratory status worsened, she was switched to high-flow nasal oxygen therapy (HFNOT) and subsequently to noninvasive ventilation with high-pressure settings: fraction of inspired oxygen (FiO_2_) 95%, inspiratory positive airway pressure (IPAP) 30 cmH_2_O, and expiratory positive airway pressure (EPAP) 14 cmH_2_O. Although all microbiological tests remained negative, empirical antimicrobial therapy was initiated, consisting of trimethoprim–sulfamethoxazole for possible *P. jirovecii* infection, together with ceftriaxone and azithromycin as broad-spectrum coverage for bacterial pneumonia. High-dose intravenous methylprednisolone was initiated early during hospitalization. Due to a lack of clinical improvement, mycophenolate mofetil was added on day five, followed by infliximab on day six.

## Follow-up and outcomes

The patient’s respiratory status continued to decline despite withdrawal of immunotherapy, escalation of immunosuppressive treatment, and progressive intensification of ventilatory support. All interventions were administered under continuous inpatient monitoring, and treatment adherence was complete. Nevertheless, the disease proved refractory to therapy, and the patient died on day seven of hospitalization, 27 days after initiating pembrolizumab.

## Discussion

This case illustrates a hyperacute and fatal presentation of CIP in a patient with newly diagnosed IPF undergoing treatment for advanced squamous-cell lung cancer. While CIP typically develops within 2–3 months of initiating ICIs, early-onset cases—defined as occurring within the first 6 weeks—have been reported and are associated with higher toxicity and poorer outcomes. Mullangi and Doraiswamy recently described a patient who developed CIP 30 days after receiving pembrolizumab, underscoring the need for early clinical vigilance—even after one or two doses (16). In the present case, symptoms emerged just 12 days after the first and only dose, representing a fulminant course of disease and raising the clinical question: What factors contributed to such an early and aggressive trajectory?

## Risk of developing CIP

Pre-existing ILD—particularly fibrotic subtypes such as IPF—is widely recognized as one of the strongest risk factors for CIP. Retrospective studies, including those by Yamaguchi et al. and Fujita et al., have consistently confirmed that patients with preexisting pulmonary fibrosis or ILD are at markedly increased risk of developing CIP after PD-1 inhibitor therapy (17, 18). This association is reinforced by the meta-analysis by Zhou et al., which integrated data from 28 studies and identified interstitial lung abnormalities, pulmonary fibrosis, and ILD as leading predictors of CIP, alongside factors such as squamous cell carcinoma histology and PD-L1 expression ≥50% (19). Many of the statistically significant risk factors identified in the meta-analysis applied to our patient and are summarized in **Table 3**.

**Table 3:** Risk factors for checkpoint inhibitor pneumonitis (19) and risk-scoring model for severe cases (13).

Risk factor	Applies to the patient	Patient status/value	OR (95% CI)
IPF diagnosis			
Interstitial lung abnormalities	No	IPF diagnosis	8.30 (4.70–14.66)
Pulmonary fibrosis	Yes	Present	6.03 (3.25–11.20)
Interstitial lung disease	Yes	Present	5.68 (3.67–8.78)
High platelet-to-lymphocyte ratio	No	80.75	3.88 (1.08–13.87)
PD-L1 expression ≥50%	Yes	≥50%	3.59 (1.23–10.50)
Thoracic radiotherapy history	No	No prior RT	3.46 (2.03–5.90)
COPD	No	Not reported	3.41 (1.45–7.99)
PD-1 inhibitor vs PD-L1	Yes	Pembrolizumab	3.10 (1.64–5.87)
Absolute eosinophil count <0.05×10^9^/L	No	0.24×10^9^/L	3.03 (1.88–4.87)
Pembrolizumab vs nivolumab	Yes	Pembrolizumab	2.89 (1.56–5.35)
Multifocal metastases ≥2 sites	Yes	Multifocal	2.77 (1.47–5.22)
Hypoalbuminemia <35 g/L	Unknown	Not assessed	2.47 (1.29–4.73)
Early-stage NSCLC (Stage III vs IV)	No	Stage IVb	2.43 (1.30–4.56)
Elevated C-reactive protein >5 mg/L	No	2.0 mg/L	2.26 (1.10–4.65)
White blood cell count >10×10^9^/L	No	8.98×10^9^/L	1.64 (1.32–2.03)
Smoking history	No	Non-smoker	1.92 (1.27–2.91)
Squamous histology	Yes	Squamous carcinoma	1.59 (1.22–2.08)
Male sex	No	Female	1.41 (1.06–1.89)
Advanced age	Yes	78 years	1.07 (1.03–1.11)
Risk-scoring model for severe cases			
ILD	Yes	5/5	4.76 (1.45–15.65)
Radiation during ICI	No	0/4	4.30 (1.77–10.45)
Pleural effusion	No	0/3	3.00 (1.48–6.08)
Emphysema	No	0/3	2.87 (1.42–5.78)
ICI monotherapy	Yes	2/2	2.44 (2.44–5.43)

CI, Confidence Interval; CIP, checkpoint inhibitor pneumonitis; COPD, Chronic Obstructive Pulmonary Disease; ICI, Immune Checkpoint Inhibitor; ILD, Interstitial Lung Disease; NSCLC, Non-Small Cell Lung Cancer; OR, Odds Ratio; PD-1, Programmed Death-1; PD-L1, Programmed Death-Ligand 1; RT, Radiotherapy. Total risk score for severe CIP: 7 (out of 17). Interpretation: Based on Deng et al., a higher total score indicates an increased risk of developing severe CIP. No official cutoffs are provided in the original study (13).

In patients with IPF, however, a fulminant respiratory decline after ICI initiation may also be interpreted as an acute exacerbation of IPF (AE-IPF). While acute exacerbation of ILD (AE-ILD) is a broader concept, encompassing acute exacerbations of various ILDs, in this case, AE-IPF is the relevant differential diagnosis. As highlighted by Zanini et al., AE-ILD and drug-induced ILD (DI-ILD) frequently overlap both clinically and radiologically, making a clear distinction challenging. In our patient, the extensive negative microbiological workup and the close temporal association with pembrolizumab initiation favor CIP, although a pembrolizumab-triggered AE-IPF cannot be fully excluded (20).

Mechanistically, IPF creates a pulmonary environment that may amplify immune-related lung injury. Nishioka et al. and Xu et al. have described how chronic epithelial damage, persistent alveolar inflammation, and impaired regulatory T-cell function contribute to a dysregulated immune milieu in IPF. In this primed context, blocking immune checkpoints may lead to exaggerated T-cell activation and cytokine-driven inflammation, increasing the risk of immune-mediated pneumonitis (2, 10). Xu et al. proposed a multifactorial model of CIP that integrates three primary domains of risk: patient-related factors (including pre-existing ILD like IPF, as well as chronic obstructive pulmonary disease and emphysema), tumor-specific features (notably lung cancer with high PD-L1 expression), and treatment-related exposures (such as ICI combined with small molecule targeted therapy or chemotherapy, and prior thoracic radiation) (2).

## Risk of severe (grade ≥3) CIP

While ILD increases CIP susceptibility, specific clinical and laboratory features predict progression to high-grade, treatment-refractory pneumonitis. Deng et al. developed a risk-scoring system incorporating five variables—ILD, thoracic radiation, pleural effusion, emphysema, and monotherapy versus combination ICI use (13). Each factor adds to a cumulative score predictive of grade ≥3 CIP. In this case, the patient had ILD and received monotherapy, yielding a score of 7 out of 17 (**Table 3**), consistent with increased vulnerability. However, precise thresholds for risk categories remain to be defined.

The patient’s laboratory profile further supports her poor prognosis. She presented with elevated inflammatory markers (CRP 81.1 mg/L, procalcitonin 0.13 ng/mL), hypoalbuminemia (30.12 g/L), anemia (hemoglobin 6.3 mmol/L), and neutrophil-to-lymphocyte ratio rising from 2 to 26—all findings suggestive of fulminant systemic inflammation. While Liu et al. focused on baseline predictors, the persistence of these markers during decompensation reflects CIP pathophysiology (21). Her lactate dehydrogenase peaked at 3585 U/L, far exceeding the >320 U/L threshold identified by Tan et al. as predictive of refractory disease, indicating severe pulmonary injury (22). Although we did not assess alveolar nitric oxide in this case, Gao et al. demonstrated that elevated levels of this marker in ILD patients may reflect subclinical pulmonary inflammation, highlighting the potential of noninvasive biomarkers to identify individuals at increased risk of pneumonitis (23).

In this case, the early onset of CIP may represent an additional poor prognostic factor. Huang et al. demonstrated that early-onset CIP is associated with a markedly higher rate of grade ≥3 events and a mortality rate of 50%, substantially exceeding that of late-onset cases (11.1%) (24). In our patient, respiratory deterioration progressed rapidly, necessitating escalation from nasal cannula to HFNOT and eventually noninvasive ventilation, illustrating the narrow therapeutic window once fulminant CIP develops.

Despite early pembrolizumab discontinuation and prompt initiation of high-dose corticosteroids, mycophenolate mofetil, and infliximab, the patient’s condition worsened. As reported by Deng et al., current immunosuppressive strategies may fail to reverse severe CIP, particularly in cases suggestive of underlying fibrosis. In their report, steroid-refractory pneumonitis was successfully managed only after the introduction of cyclosporine, highlighting its potential as an effective second-line treatment in selected patients (25).

All laboratory values and risk factors refer to baseline measurements prior to the initiation of pembrolizumab.

## Clinical implications and future directions

Given the substantial risk of fatal CIP in patients with fibrotic ILD, there is a growing need for refined immunotherapy strategies. Emerging approaches supported by recent literature include:

- Clinical risk-scoring models that integrate laboratory markers, comorbidities, and radiologic features to predict CIP susceptibility.
- Pre-treatment assessment of inflammatory and hematologic markers — including absolute eosinophil count, CRP, hemoglobin, neutrophil-to-lymphocyte ratio, platelet-to-lymphocyte ratio, serum albumin, and white blood cell count — may help identify patients with immune-primed lungs (19, 21).
- Emerging biomarker research, such as studies investigating alveolar nitric oxide (23).
- Concurrent or prophylactic antifibrotic therapy, using agents such as pirfenidone or nintedanib, which possess both antifibrotic and immunomodulatory properties (26, 27).
- Early cytokine blockade, particularly IL-6 inhibition, has been proposed as a targeted strategy to interrupt key inflammatory pathways in IPF and CIP (28).
- Systematic data collection with national registries and prospective studies is essential to refine diagnostic criteria, identify predictive biomarkers, and evaluate long-term outcomes in CIP—gaps that remain largely unmet in current clinical practice.

Implementation of these strategies will require a multidisciplinary approach. Pre-treatment evaluation by oncology, pulmonology, and radiology teams should become routine for patients with fibrotic ILD. Enhanced post-treatment surveillance—including early imaging and symptom monitoring—may improve the detection of CIP at its earliest and most manageable stage.

As ICIs are increasingly adopted across malignancies, embedding individualized risk assessment and stratified care into clinical workflows will reduce harm and improve outcomes in this vulnerable patient population.

## Conclusions

This case underscores the potential for rapid-onset, fulminant CIP in patients with underlying ILD, particularly IPF. Notably, life-threatening CIP occurred after a single dose of pembrolizumab, highlighting the severity and unpredictability of immune-related toxicity in this high-risk population. Standard immunosuppressive therapies proved insufficient to reverse clinical deterioration, emphasizing the urgent need for prospective trials investigating prophylactic antifibrotic strategies and early cytokine blockade. As ICIs continue to expand across cancer types, validated, clinically usable risk stratification tools—ideally supported by digital platforms—will be essential. Ultimately, multidisciplinary evaluation, individualized risk assessment, and tailored surveillance are critical to optimizing the safety of immunotherapy in patients with preexisting fibrotic lung disease.

## Patient perspective

Although the patient was unable to share her perspective due to the rapid progression of her illness, her family remained closely involved in her care. They were present at her bedside throughout her hospitalization and expressed hope for her recovery. Following her passing, they acknowledged the inevitability of her condition and expressed gratitude for the attentive care and open communication they received.

## Data Availability

The original contributions presented in the study are included in the article/**Supplementary Material**. Further inquiries can be directed to the corresponding author.

## Supplementary material

Supplementary Table 1 Results of multiplex respiratory PCR panel (BioFire Respiratory Panel 2.1 plus, nested PCR) (performed 15.05 2025).
