# Supplementary material for: Fatal early-onset checkpoint inhibitor pneumonitis in a patient with advanced squamous-cell lung cancer with underlying pulmonary fibrosis: a case report and review of the literature
Source: Front Oncol. 2025 Nov 4;15:1672093. doi: 10.3389/fonc.2025.1672093 (PMC12623183; doi:10.3389/fonc.2025.1672093)
Supplement: Supplementary Table 1 — Results of multiplex respiratory PCR panel (BioFire Respiratory Panel 2.1 plus, nested PCR) (performed 15.05 2025). [file Table1.docx]

# **Supplementary Table 1. Results of multiplex respiratory PCR panel (BioFire Respiratory Panel 2.1 plus, nested PCR) (performed 15.05 2025).**

| **Pathogen** | **Result** |
| --- | --- |
| Influenza A virus RNA | Negative |
| Influenza B virus RNA | Negative |
| Parainfluenza virus type 1 RNA | Negative |
| Parainfluenza virus type 2 RNA | Negative |
| Parainfluenza virus type 3 RNA | Negative |
| Parainfluenza virus type 4 RNA | Negative |
| Coronavirus NL63 RNA | Negative |
| Coronavirus 229E RNA | Negative |
| Coronavirus OC43 RNA | Negative |
| Coronavirus HKU1 RNA | Negative |
| MERS-CoV RNA | Negative |
| SARS-CoV-2 RNA | Negative |
| Human metapneumovirus RNA | Negative |
| Rhinovirus/enterovirus RNA | Negative |
| Respiratory syncytial virus (RSV) type A/B RNA | Negative |
| Adenovirus DNA | Negative |
| Mycoplasma pneumoniae DNA | Negative |
| Chlamydophila pneumoniae DNA | Negative |
| Bordetella pertussis DNA | Negative |
| Bordetella parapertussis DNA | Negative |
